# Thallium-201 Imaging in Intact Olfactory Sensory Neurons with Reduced Pre-Synaptic Inhibition In Vivo

**DOI:** 10.1007/s12035-020-02078-y

**Published:** 2020-08-20

**Authors:** Hideaki Shiga, Hiroshi Wakabayashi, Kohshin Washiyama, Tomohiro Noguchi, Tomo Hiromasa, Sadaharu Miyazono, Masami Kumai, Kazuma Ogawa, Junichi Taki, Seigo Kinuya, Takaki Miwa

**Affiliations:** 1 Department of Otorhinolaryngology, Kanazawa Medical University, Uchinadamachi, Kahokugun, Ishikawa 920-0293 Japan; 2 Department of Nuclear Medicine, Graduate School of Medical Sciences, Kanazawa University, Kanazawa, Ishikawa 920-8640 Japan; 3 Advanced Clinical Research Center, Fukushima Global Medical Science Center, Fukushima Medical University, Fukushima, 960-1295 Japan; 4 Department of Sensory Physiology, Asahikawa Medical University, Asahikawa, 078-8510 Japan; 5 Institute for Frontier Science Initiative, Kanazawa University, Kanazawa, Ishikawa 920-1192 Japan

**Keywords:** Olfactory dysfunction, Dopaminergic interneuron, Tyrosine hydroxylase, Action potential, Olfactory transport, Rotenone

## Abstract

In this study, we determined whether the ^201^Tl (thallium-201)-based olfactory imaging is affected if olfactory sensory neurons received reduced pre-synaptic inhibition signals from dopaminergic interneurons in the olfactory bulb in vivo. The thallium-201 migration rate to the olfactory bulb and the number of action potentials of olfactory sensory neurons were assessed 3 h following left side nasal administration of rotenone, a mitochondrial respiratory chain complex I inhibitor that decreases the number of dopaminergic interneurons without damaging the olfactory sensory neurons in the olfactory bulb, in mice (6–7 animals per group). The migration rate of thallium-201 to the olfactory bulb was significantly increased following intranasal administration of thallium-201 and rotenone (10 μg rotenone, *p* = 0.0012; 20 μg rotenone, *p* = 0.0012), compared with that in control mice. The number of action potentials was significantly reduced in the olfactory sensory neurons in the rotenone treated side of 20 μg rotenone-treated mice, compared with that in control mice (*p* = 0.0029). The migration rate of thallium-201 to the olfactory bulb assessed with SPECT-CT was significantly increased in rats 24 h after the left intranasal administration of thallium-201 and 100 μg rotenone, compared with that in control rats (*p* = 0.008, 5 rats per group). Our results suggest that thallium-201 migration to the olfactory bulb is increased in intact olfactory sensory neurons with reduced pre-synaptic inhibition from dopaminergic interneurons in olfactory bulb glomeruli.

**Electronic supplementary material:**

The online version of this article (10.1007/s12035-020-02078-y) contains supplementary material, which is available to authorized users.

## Introduction

Detecting the mechanisms underpinning olfactory dysfunction is challenging, owing to the difficulty in directly viewing the connectivity of peripheral olfactory nerves using current magnetic resonance imaging (MRI). To date, we have visualized olfactory nerves in healthy volunteers and olfactory-impaired patients using a novel olfactory nerve tracer, radioisotope ^201^Tl (thallium-201) [1, 2]. Thallium-201-based olfactory imaging is referred to as olfactory scintigraphy.

The radioisotope thallium-201 is transported within olfactory neural tracts after nasal administration in rodents [3]. This transport is significantly decreased by transecting olfactory nerve fibers and correlates with odor detection ability in mice [4, 5]. Thallium can readily substitute potassium at the sodium/potassium (Na+/K+)-membrane adenosine triphosphatase (ATPase) activation sites [6]. Furthermore, nasally administered thallium-201 may be transported into olfactory nerve cells as a substitute for potassium.

Periglomerular cells are dopaminergic inhibitory interneurons in the glomerular layer of the olfactory bulb. The inhibitory interneurons in the olfactory bulb increase the dynamic range of information transfer from olfactory receptor neurons to olfactory bulb neurons [7] and maintain odor discriminating ability [8].

The glomerular layer of the olfactory bulb is damaged in rats treated with diethyldithiocarbamate, a toxic agent that induces the degeneration of the olfactory epithelium [9]. The olfactory bulb volume is reduced in patients with olfactory loss, which suggests that the number of dopaminergic inhibitory interneurons in the olfactory bulb of these patients is also reduced [10, 11]. Inhibition of olfactory sensory neurons is mediated by suppression of pre-synaptic calcium influx with GABA and dopamine released from dopaminergic interneurons in olfactory bulb of the vertebrates [12, 13]. However, it remains unclear whether thallium-201 migration to the olfactory bulb is affected by the pre-synaptic inhibition of olfactory sensory neurons from dopaminergic interneurons in olfactory bulb glomeruli of olfactory-impaired patients with a reduced olfactory bulb volume.

Rotenone, a mitochondrial respiratory chain complex I inhibitor, has been commonly used as a pesticide in agriculture [14]. In mice, nasal administration of rotenone decreases the number of dopaminergic interneurons in the olfactory bulb without decreasing the expression of olfactory marker proteins in the olfactory bulb [15].

In this study, following nasal administration of rotenone in rodents, we investigated thallium-201 migration to the olfactory bulb with a reduced number of dopaminergic interneurons in the olfactory bulb and intact olfactory sensory neurons. The aim of our study was to determine whether thallium-201 migration to the olfactory bulb is affected if the intact olfactory sensory neurons received reduced pre-synaptic inhibition signal from the dopaminergic interneurons in the olfactory bulb in vivo.

## Materials and Methods

### Experimental Procedures

#### Materials

Eight-week-old male ICR mice (CLEA Japan, Inc., Tokyo, Japan) and 8-week-old male Wistar rats (SLC Japan, Inc., Hamamatsu, Japan) were housed in a 22–26 °C air-conditioned room with a 12-h light:dark cycle. Food (CLEA Japan, Inc., Tokyo, Japan) and water were provided ad libitum. Rats were selected, as opposed to mice, for in vivo imaging with SPECT-CT because the head of a mouse is too small for imaging the migration of thallium-201 to the olfactory bulb with SPECT-CT.

All animal experimental procedures were approved by the animal experiment committees of Kanazawa Medical University (Ishikawa, Japan; number: 2016-85), Kanazawa University (Ishikawa, Japan; number: AP-153663), and Asahikawa Medical University (Asahikawa, Japan; number: 17053).

#### Nasal Administration of Thallium Chloride [^201^Tl]TlCl and Rotenone Solution in Mice

[^201^Tl] Thallium (I) chloride (^201^Tl, 74 MBq/mL) was administered into the left nasal cavity of each mouse via a microinjection pipette. Sneezing was prevented with anesthesia (intraperitoneal administration of a mixed solution of medetomidine [0.75 μg/g], midazolam [4 μg/g], and butorphanol [5 μg/g]) in phosphate-buffered saline (PBS). Mice were awakened with the intraperitoneal administration of atipamezole 30 min after the nasal administration of the solution. The thallium-201 solution was obtained from Nihon Medi-Physics (Tokyo, Japan). Ten microliters of thallium-201 in the vehicle control (1% dimethyl sulfoxide [DMSO] in PBS, *N* = 6), 10 μg rotenone (Sigma, Saint Louis, MI, USA, *N* = 6), or 20 μg rotenone (*N* = 6) was administered into the left nasal cavity of each normal mouse under anesthesia. Three hours after the thallium-201 nasal administration, mice were euthanized under anesthesia.

Tissue samples were obtained from the left olfactory bulb and left nasal turbinate. After the weight measurements, sample radioactivity was measured with gamma spectrometry using the Auto Well Gamma System (model ARC-380; Hitachi, Tokyo, Japan). The rate of thallium-201 migration to the olfactory bulb was calculated as the uptake of thallium-201 per gram of wet weight (% dose/g) in the left olfactory bulb divided by the uptake of thallium-201 per gram of wet weight (% dose/g) in the left nasal turbinate [4, 5].

#### Immunohistochemistry for Dopaminergic and Olfactory Sensory Neurons

We analyzed the expression of tyrosine hydroxylase to ascertain the location of dopaminergic interneurons in the olfactory bulb by immunohistochemical staining since tyrosine hydroxylase is a specific marker of dopaminergic interneurons [15]. The bilateral olfactory bulbs and nasal turbinates were also assessed using immunohistochemical staining for olfactory marker protein expression. Three hours after undergoing left nasal administration of 20 μg rotenone in 10 μL of 1% DMSO solution, normal mice (*N* = 6) were perfused with physiological saline, and fixed with 4% paraformaldehyde under anesthesia (the intraperitoneal administration of a mixed solution containing medetomidine [0.75 μg/g], midazolam [4 μg/g], and butorphanol [5 μg/g]). The head was dissected, and facial bones were removed. Following overnight fixation with 4% paraformaldehyde, the bilateral olfactory bulbs and nasal turbinates were resected. Sample tissues were treated in 20% sucrose solution (Sigma, Osaka, Japan) and embedded in paraffin. Samples of nasal turbinates were separated from the olfactory bulbs and were decalcified for 7 days at 4 °C with a commercially based decalcification solution (KC-X; FALMA, Tokyo, Japan) before paraffin embedding. The samples were sliced into 3-μm sections and mounted on slides for immunohistochemical staining.

Sections of the bilateral olfactory bulbs were deparaffinized with xylene and rehydrated through a graded alcohol series. For the staining of tyrosine hydroxylase, after blocking and antigen retrieval (90 °C for 30 min), the sections were incubated with anti-tyrosine hydroxylase antibody (1:2000 dilution; Abcam, Tokyo, Japan) in an antibody diluent (Dako Cytomation, Glostrup, Denmark) for 1 h at 24 °C. After washing with PBS, each section was incubated for 30 min at 24 °C with DyLight 649-conjugated anti-chicken immunoglobulin G (IgG) (H&L) antibody (1:5000 dilution; Rockland, Limerick, PA, USA), and then washed in PBS. After development, the sections were mounted.

For the staining of olfactory marker protein (a marker of mature olfactory neurons), after blocking and antigen retrieval (90 °C for 30 min), each section was incubated for 1 h at 24 °C with anti-olfactory marker protein antibody (1:100 dilution; FUJIFILM Wako, Osaka, Japan) in an antibody diluent, and then washed in PBS. Sections were incubated for 30 min at room temperature with Alexa Fluor 555-conjugated anti-goat IgG (H&L) antibody (1:200 dilution; Life Technologies, Carlsbad, CA, USA), and then washed in PBS. After developing, the sections were mounted.

The sections of nasal turbinates were also stained for olfactory marker protein. After development, the sections were lightly counterstained with 4′,6-diamidino-2-phenylindole solution (DAPI) (Vector TrueVIEW™; Vector Laboratories, CA, USA) and mounted.

For the negative control, the antibody diluent was applied instead of the primary antibody solution. Slides were observed under a fluorescence microscope (BZ-X700; Keyence Corporation, Osaka, Japan). Immunofluorescence intensity for TH and olfactory marker protein expressions in the images (× 40) was assessed with an analysis application measurement module (BZ-H3M; Keyence) in the right and left olfactory bulb of each sample. The number of olfactory marker protein-positive cells was manually counted in the 250 μm basal membrane length olfactory epithelium of the middle part each side nasal septum. The histological analysis was separately performed by two investigators (HS and MK) in a blinded manner for each sample image, and the average of scores was used for statistical analysis.

#### Electrophysiological Analysis of Olfactory Sensory Neurons

Three hours after the intranasal administration of 20 μg rotenone or the vehicle (1% DMSO) into the left nasal cavity, ICR male mice (8 weeks old) were deeply anesthetized with an intraperitoneal injection of pentobarbital sodium (150 mg/kg), followed by sevoflurane inhalation. To protect tissue from excitotoxic damage, the animals were treated with cardiac perfusion with ice-cold sucrose-based Ringer’s solution (in mM: 234 sucrose, 2.5 potassium chloride [KCl], 26 sodium bicarbonate [NaHCO_3_], 1.25 sodium dihydrogen phosphate [NaH_2_PO_4_], 0.5 calcium chloride [CaCl_2_], 10 magnesium chloride [MgCl_2_], and 11 glucose, pH 7.4) oxygenated with 95% oxygen (O_2_) and 5% carbon dioxide (CO_2_) mixed gas before decapitation.

Electrophysiology of the olfactory sensory neurons was conducted, as previously described [16]. In brief, the inside of the nasal cavity of mice was disclosed by splitting the head along the midline. A sheet of olfactory epithelium in the range between the septum and dorsal region of the nasal cavity was peeled away under a stereomicroscope. The remainder of the nasal turbinates was trimmed. The somata of olfactory sensory neurons were exposed for patch clamp recordings by making a shallow incision on the apical surface of the sensory epithelia with the tip of a 25-gauge needle. The epithelia were incubated in normal Ringer’s solution (in mM: 125 NaCl, 2.5 KCl, 26 NaHCO_3_, 1.25 NaH_2_PO_4_, 2 CaCl_2_, 1 MgCl_2_, 11 glucose, pH 7.4) saturated with O_2_/CO_2_ gas at 37 °C for 30 min and equilibrated in the same solution at room temperature (24–26 °C) until use.

The epithelial preparation was placed on the glass bottom of a recording chamber filled with normal Ringer’s solution saturated with O_2_/CO_2_ gas and fixed by a U-shaped weight. Individual cells were observed by infrared differential interference contrast video microscopy through an E600FN microscope (Nikon, Tokyo, Japan) with a water-immersion 40× objective lens. Glass electrodes were filled with a potassium (K)-gluconate intracellular solution (in mM: 140 K-gluconate, 2 MgCl_2_, 2 adenosine triphosphate [Na_2_ATP], 0.5 ethylene glycol tetra-acetic acid/potassium hydroxide [EGTA/KOH], 10 4-(2-hydroxyethyl)-1-piperazineethanesulfonic acid [HEPES], pH 7.2/KOH). The electrode resistance was 12 ± 0.67 MΩ (*N* = 32). The seal resistance achieved with glass electrode tips on the cell surface was 9.7 ± 1.5 GΩ (*N* = 28). Series resistance from formation of whole-cell patch configuration was 43 ± 2.6 MΩ (*N* = 32).

Membrane potential and currents were acquired using an Axopatch 200B amplifier (Molecular Devices, Sunnyvale, CA, USA) with a 10-KHz low-pass filter. They were digitized using a Digidata 1320A digitizer (Molecular Devices) controlled on pClamp software (Molecular Devices) at a sampling frequency of 20 kHz. The capacitive current and compensation of series resistance were manually subtracted by using a built-in circuit of the amplifier in the range without overcompensation. Liquid junction potentials were estimated at 15 mV and were adjusted in the analyses.

#### Single-Photon Emission Computed Tomography/Computed Tomography Analysis in Normal Rats Treated with Rotenone

Twenty-four hours after left nasal administration of 50 μL of ^201^TlCl solution with the control vehicle (1% DMSO in PBS) or with 100 μg rotenone, normal rats were examined using SPECT-CT (versatile emission computed tomography/computed tomography; MILabs, Utrecht, Netherlands) under anesthesia. Data were acquired in list mode and dual photopeak windows (76 keV, 28% width and 168 keV, 20% width) were set after the acquisition. Triple energy window scatter correction was employed in this experiment. Data were reconstructed using pixel-based order-subsets expectation maximization in 32 subsets and 8 iterations using the method, which was previously shown [17], with correction for attenuation on computed tomography.

The obtained SPECT images were analyzed with the public domain AMIDE imaging software (version 1.01) [18]. Volumes of interest were manually placed for the olfactory nerve and nasal cavity. The ^201^Tl migration rate to the olfactory bulb was calculated, based on the following formula: $201-\mathrm{Tl}\ \mathrm{migration}\ \mathrm{to}\ \mathrm{olfactory}\ \mathrm{bulb}\ \mathrm{rate}\ \left(\%\right)=\frac{\mathrm{total}\ \mathrm{counts}\ \mathrm{in}\ \mathrm{the}\ \mathrm{olfactory}\ \mathrm{bulb}}{\ \mathrm{total}\ \mathrm{counts}\ \mathrm{in}\ \mathrm{the}\ \mathrm{nasal}\ \mathrm{cavity}}\times 100$. The imaging analysis was separately performed by two investigators (HW and TH) in a blinded manner for each image, and the average of scores was used for statistical analysis.

### Statistical Analysis

We compared mean values using a Mann-Whitney *U* test (Prism 6, GraphPad, San Diego, CA, USA). All *p* values were two-tailed. Electrophysiological data were analyzed with one-way ANOVA followed by Scheffe’s multiple comparisons using Excel software (Microsoft, Redmond, WA, USA) with Visual Basic-based macro programs. Ward’s method, a hierarchical cluster analysis, was employed for classifying olfactory sensory neurons with standardized values of conductance for voltage-gated Na^+^ (G_Na_) and K^+^ (G_K_) currents. In multiple comparisons for proportion tests, α-error rate was corrected with Ryan’s nominal significance level. Data values are shown as mean ± 95% confidence intervals unless otherwise noted. A *p* value of < 0.05 was considered statistically significant.

## Results

### Nasal Administration of ^201^TlCl and Rotenone Solution in Mice

**Fig. 1:**
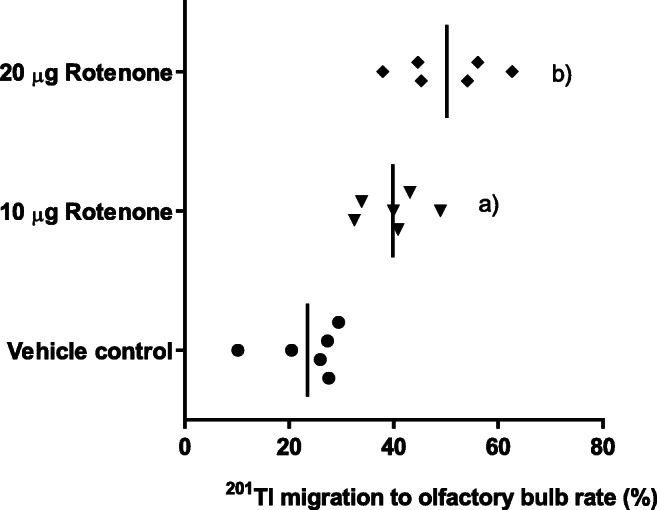
The thallium-201 migration rate to the olfactory bulb was significantly increased in normal mice 3 h after the left intranasal administration of ^201^Tl and rotenone ([a] 10 μg rotenone, *p* = 0.0012, *N* = 6; [b] 20 μg rotenone, *p* = 0.0012, *N* = 6) compared with the rate after treatment with ^201^Tl and the vehicle control (1% DMSO in PBS, *N* = 6). The bars indicate the mean

Three hours after intranasal administration of ^201^Tl and rotenone, the thallium-201 migration rate to the olfactory bulb was significantly increased in normal mice (Mann-Whitney test: 10 μg rotenone, *p* = 0.0012, the mean ± the S.D. = 39.8 ± 6.1, *N* = 6; 20 μg rotenone, *p* = 0.0012, the mean ± the S.D. = 50.1 ± 9.1, *N* = 6; Fig. 1), compared with the rate after treatment with ^201^Tl and the vehicle control (1% DMSO in PBS, the mean ± the S.D. = 23.5 ± 7.2, *N* = 6). We selected 20 μg rotenone as the treatment in subsequent experiments.

### Immunohistochemistry for Dopaminergic and Olfactory Sensory Neurons

**Fig. 2:**
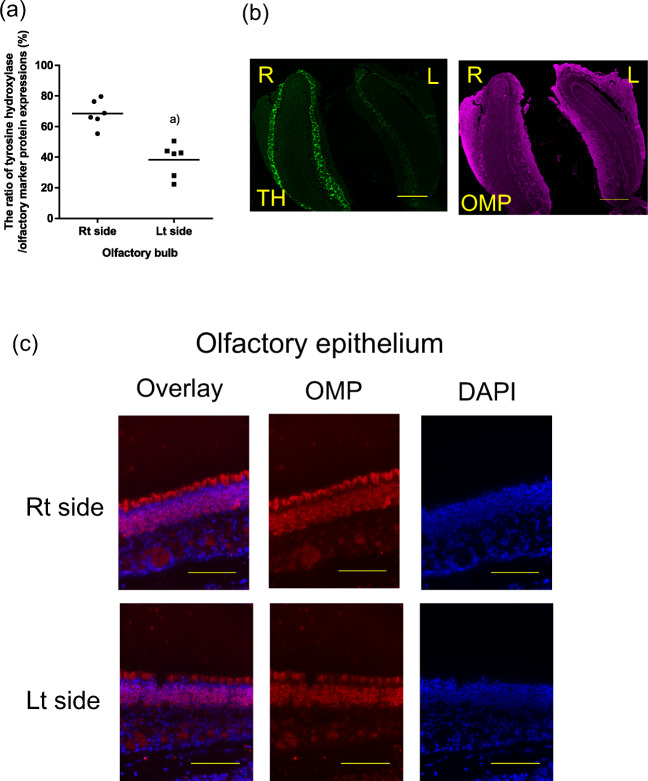
(a) The ratio of tyrosine hydroxylase (TH) expression divided by the olfactory marker protein (OMP) expressions in the left olfactory bulb was significantly decreased 3 h after the left intranasal administration of 20 μg rotenone, compared with the ratio in the right olfactory bulb in the mice ([a] *p* = 0.0002, *N* = 6/group). The bars indicate mean. (b) Representative axial images of TH (green) and OMP (magenta) expressions determined with immunohistochemical staining in the olfactory bulb of mice treated with 20 μg rotenone. R, right side; L, left side. TH expression was mostly observed in the glomerular layer of the olfactory bulb. The bars indicate 500 μm. (c) Representative images of olfactory marker protein (OMP; red) and DAPI (blue) expression determined with immunohistochemical staining in the olfactory epithelium of mice 3 h after the left intranasal administration of 20 μg rotenone. Scale bars indicate 100 μm. Rt, right; Lt, left

The ratio of tyrosine hydroxylase expression divided by the olfactory marker protein expression in the left olfactory bulb was significantly decreased 3 h after the left intranasal administration of 20 μg rotenone, compared with the ratio in the right olfactory bulb in the mice (Mann-Whitney test, *p* = 0.0002, 6 mice per group; Fig. 2a). Representative axial images of tyrosine hydroxylase and olfactory marker protein expressions, determined using immunohistochemical staining in the olfactory bulb of mice treated with 20 μg rotenone, are in Fig. 2b. Tyrosine hydroxylase expressions were observed predominantly in the glomerular layer of the olfactory bulb.

The number of cells positive with olfactory marker protein expression in the left olfactory epithelium was not significantly changed 3 h following left intranasal administration of 20 μg rotenone, compared with those in the right olfactory epithelium in the mice. The number of cells positive with olfactory marker protein expression per 250 μm of basal layer length in the middle part of each bilateral nasal septal olfactory epithelium was as follows (presented as the mean ± the S.D.): right side, 111.2 ± 26.5; left side, 110.2 ± 24.9 (Mann-Whitney test, *p* = 0.73, 6 animals per group). Representative images of olfactory marker protein expression, determined using immunohistochemical staining in the olfactory epithelium of mice treated with 20 μg rotenone, are shown in Fig. 2c.

### Electrophysiological Analysis of Olfactory Sensory Neurons

Electrophysiological recordings were performed in normal Ringer’s solution containing no rotenone. Firing properties were compared between olfactory sensory neurons in the right olfactory epithelium isolated from vehicle control mice (Co_Rt_), left epithelium from the control (Co_Lt_), right epithelium from rotenone administration mice (Ro_Rt_), and left epithelium from the rotenone mice (Ro_Lt_). Vehicle (1% DMSO) or 20 μg rotenone was administered to the left nasal cavity of mice as described in the material and methods section.

**Fig. 3:**
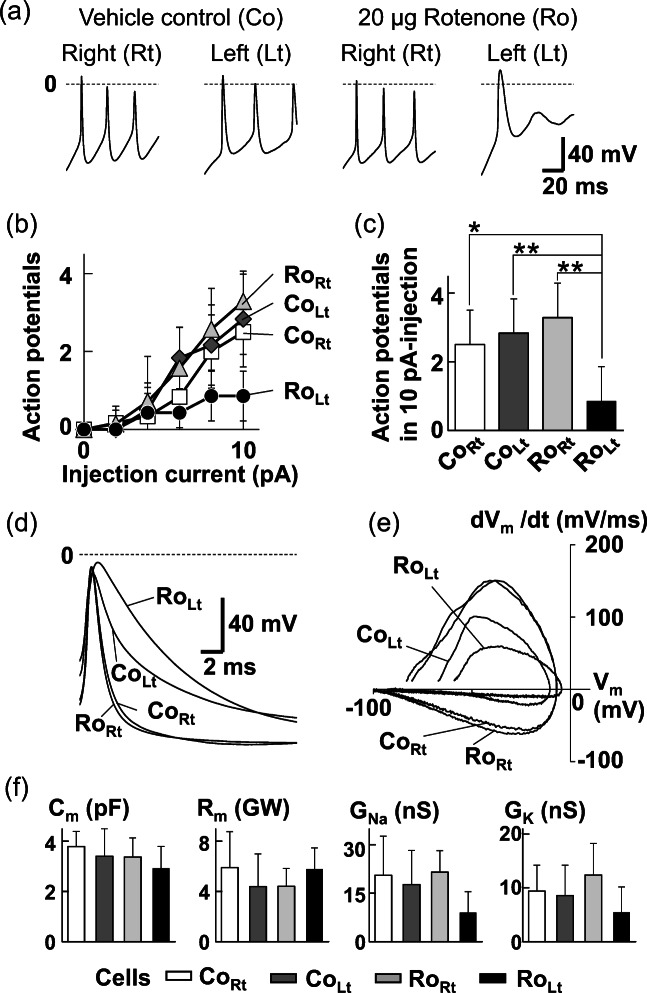
Excitability of olfactory sensory neurons 3 h after the left intranasal administration of 20 μg rotenone or the vehicle control (1% DMSO) in normal mice. (a) Typical firing of olfactory sensory neurons after intranasal administration of 20 μg rotenone or vehicle control. Action potentials were elicited by 10-pA current injections. (b) Relationships between number of action potentials and amplitude of injection currents during 100-ms step pulses. Squares and diamonds: olfactory sensory neurons on the right and left olfactory epithelium in the control mice (CoRt, *N* = 6 and CoLt, *N* = 6), respectively; triangles and circles: olfactory sensory neurons on the right and left olfactory epithelium in 20 μg rotenone-treated mice (RoRt, *N* = 7 and RoLt, *N* = 7), respectively. (c) Significant reduction of the number of action potentials by the intranasal administration of rotenone. Data points are shown in Fig. 3b at 10 pA. **p < 0.01 and *p < 0.05 by Scheffe’s multiple comparisons following one-way ANOVA. (d) Different form of action potentials in ensemble average: CoRt (*N* = 3), CoLt (*N* = 6), RoRt (*N* = 6), RoLt (*N* = 4). (e) Phase-plane plot for the action potentials shown in Fig. 3d. The ensemble average of the time derivative of the action potential (dVm/dt) is plotted to the average value of Vm of each time point. The time derivative of the membrane potentials shows current density through voltage-gated channels (Iionic/Cm = − dVm/dt). (f) Comparison of membrane properties between CoRt (*N* = 7), CoLt (*N* = 8), RoRt (*N* = 9), and RoLt (*N* = 8). Cm, membrane capacitance; Rm, membrane resistance; GNa and GK, conductance for voltage-gated Na+ and K+ currents, respectively. No statistical significance was shown by one-way ANOVA in all comparison

First, olfactory sensory neurons generated action potentials in response to 10-pA current step pulses for 100 ms although the Ro_Lt_ cells ceased firing spontaneously despite the continuous current injection (Fig. 3a). Increases in numbers of action potentials with increasing current injection were suppressed in the Ro_Lt_ cells (Fig. 3b). The number of action potentials elicited in the Ro_Lt_ cells by 10-pA current stimulus was significantly reduced (*p* = 0.0147, 0.0029, and 1.9 × 10^−4^; Scheffe’s multiple comparison) compared with Co_Rt_, Co_Lt_, and Ro_Rt_, respectively (Fig. 3c). Shapes of single action potentials produced by the Ro_Lt_ cells were higher and broader than that by the other cells (Fig. 3d). Furthermore, rising and decaying slopes in the action potentials decreased in the Ro_Lt_ cells (Fig. 3e). The characteristics of the action potentials of the Ro_Lt_ cells imply delay of repolarization due to decrease in voltage-gated currents. Mean values of membrane capacitance (C_m_), membrane resistance (R_m_), and conductance of voltage-gated Na^+^ (G_Na_) and K^+^ (G_K_) currents were compared between Co_Rt_, Co_Lt_, Ro_Rt_, and Ro_Lt_ (Fig. 3f). No statistical significance (*p* > 0.05) was shown by one-way ANOVA in all comparisons.

**Fig. 4:**
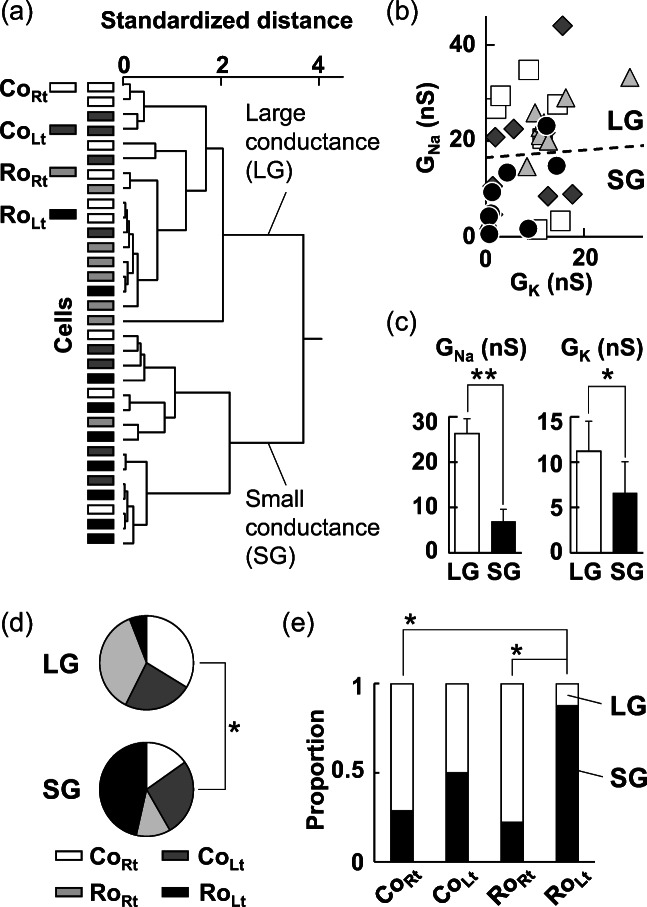
Clustering of olfactory sensory neurons 3 h after the left intranasal administration of 20 μg rotenone or the vehicle control (1% DMSO) in normal mice. (a) Dendrogram showing olfactory sensory neurons clustered based on the values of GNa and GK. Examined cells comprising of CoRt (*N* = 7), CoLt (*N* = 8), RoRt (*N* = 9), and RoLt (*N* = 8) were divided into large conductance (LG) cells of 17 and small conductance (SG) cells of 15. (b) Distribution of LG and SG cells on the GNa-GK plane. Dashed line indicates a statistically significant boundary between LG and SG, estimated by linear discriminant analysis (Wilk’s *Λ*, 0.25; *p* = 1.4 × 10^–9^). Squares: CoRt; diamonds: CoLt; triangles: RoRt; circles: RoLt. (c) Comparisons of means for GNa (left) and GK (right) between LG and SG. **p < 0.01; *p < 0.05 Welch’s *t* test. (d) Significant difference in proportion of olfactory sensory neurons between LG and SG (Fisher’s exact test, *p* = 0.042). (e) Comparing proportion of olfactory sensory neurons classified into LG and SG between CoRt, CoLt, RoRt, and RoLt. *p < Ryan’s nominal significance level. The significance level of *α* is set at 0.05 on the whole

Subsequently, olfactory sensory neurons were classified based on the values of G_Na_ and G_K_ by using cluster analysis and were divided into two groups of large conductance (LG) and small conductance (SG) cells (Fig. 4a). LG cells showing larger G_Na_ consist of 5 Co_Rt_ cells, 4 Co_Lt_ cells, 7 Ro_Rt_ cells, and 1 Ro_Lt_ cell (total of 17 cells) and SG cells showing smaller G_Na_ consist of 2 Co_Rt_ cells, 4 Co_Lt_ cells, 2 Ro_Rt_ cells, and 7 Ro_Lt_ cells (total of 15 cells) (Fig. 4b). The mean value of G_Na_ in LG cells (26 ± 3.3 nS) is significantly larger (*p* = 1.1 × 10^−10^, Welch’s *t* test) than that in SG cells (6.9 ± 2.7 nS). In addition, G_K_ of LG cells (11 ± 3.3 nS) is significantly larger (*p* = 0.049, Welch’s *t* test) than that of SG cells (6.6 ± 3.4 nS) (Fig. 4c). C_m_ showed a significant difference (*p* = 0.042, Welch’s *t* test) between LG cells (3.7 ± 0.56 pF) and SG cells (3.0 ± 0.46 pF) but R_m_ showed no significant difference (*p* = 0.80, Welch’s *t* test) between LG cells (5.2 ± 1.3 GΩ) and SG cells (5.0 ± 1.4 GΩ).

Proportion of cells composing each group is significantly different (p = 0.042, Fisher’s exact test) between LG and SG cells (Fig. 4d). Particularly, proportion of Ro_Lt_ cells classified into SG cells (7 of 8 cells, 88%) is significantly larger (*p* = 0.011 and *p* = 3.6 × 10–3, Ryan’s multiple comparison) than that of Co_Rt_ (2 of 7 cells, 29%) and Ro_Rt_ (2 of 9 cells, 22%) cells, respectively (Fig. 4e). These results suggest that the rotenone administration to nasal cavity increases occurrence ratio of smaller G_Na_ and G_K_ in olfactory sensory neurons on the drug administered side.

### SPECT-CT Analysis in Normal Rats Treated with Rotenone

**Fig. 5:**
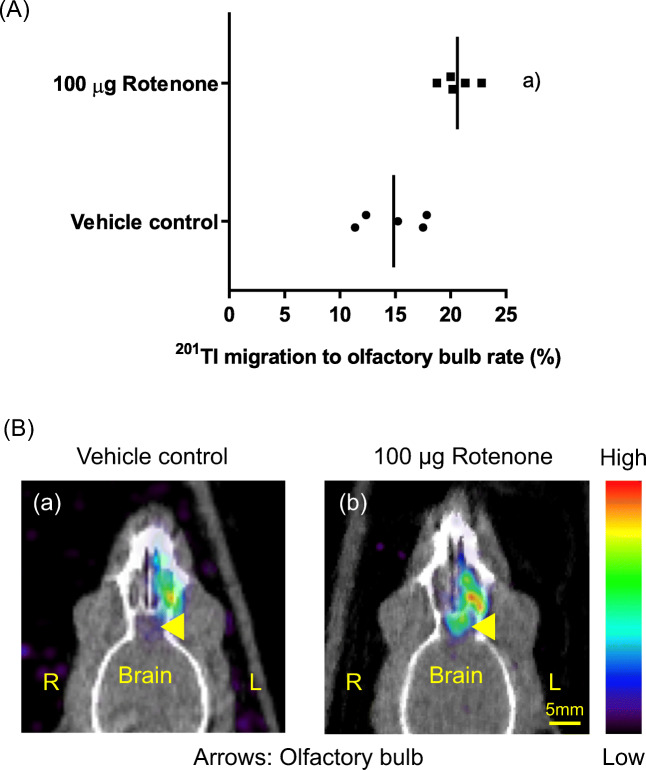
(a) The thallium-201 migration rate to the olfactory bulb assessed with SPECT-CT was significantly increased in normal rats 24 h after the left intranasal administration of ^201^Tl and 100 μg rotenone, compared with the rate after treatment with ^201^Tl and the vehicle control (1% DMSO in PBS) ((a) *p* = 0.008, *N* = 5/group). The bars indicate the mean. (b) Representative axial images of SPECT-CT 24 h after nasal administration of ^201^Tl with vehicle control (a) or 100 μg rotenone (b). R, right side; L, left side

We assessed SPECT-CT images obtained 24 h following nasal administration of ^201^Tl in the rats. The thallium-201 migration rate to the olfactory bulb, as assessed with SPECT-CT, was significantly increased in normal rats 24 h following intranasal administration of ^201^Tl and 100 μg rotenone, compared with the rate following treatment with ^201^Tl and vehicle control (1% DMSO in PBS) (Fig. 5a, Mann-Whitney test, *p* = 0.008, 5 rats per group). An increase in the thallium-201 migration rate to the olfactory bulb in rats treated with 100 μg rotenone, compared with the rate in the control rats, was detected with in vivo imaging (Fig. 5b).

## Discussion

In this study, we aimed to determine whether thallium-201 migration to the olfactory bulb is affected by pre-synaptic inhibition of olfactory sensory neurons from the dopaminergic interneurons in the olfactory bulb in vivo*.* To accomplish this, we investigated thallium-201 migration to the olfactory bulb in mice with reduced numbers of dopaminergic interneurons in the olfactory bulb and mature olfactory sensory neurons without significant damage, after the nasal administration of rotenone.

The thallium-201 migration rate to the olfactory bulb was significantly increased after the intranasal administration of ^201^Tl and rotenone in mice. Dopaminergic neurons exhibit high sensitivity to rotenone-induced cytotoxicity mediated by reactive oxygen species [14, 15]. In the current study, tyrosine hydroxylase expression, which is a specific marker of dopaminergic interneurons in the glomerular layer of the olfactory bulb, was significantly decreased after intranasal administration of rotenone in mice. Periglomerular cells are dopaminergic inhibitory interneurons in the olfactory bulb. The decrease in tyrosine hydroxylase expression in the glomerular layer of the olfactory bulb after the intranasal administration of rotenone suggested that the numbers of periglomerular cells were reduced in mice treated with rotenone.

In this study, mice olfactory neurons were not significantly damaged 3 h following 20 μg rotenone treatment, thereby promoting nasal thallium-201 migration to the olfactory bulb, while nasal thallium-201 migration to the olfactory bulb is reduced in patients with impaired olfaction due to upper respiratory tract infections, compared with ^201^Tl migration in healthy volunteers [2]. Histopathological analyses have shown damage to the olfactory epithelium in patients with impaired olfaction due to upper respiratory tract infection [19]. Decreased olfactory bulb volume has been reported in patients with impaired olfaction caused by upper respiratory tract infections [2]. Therefore, a disconnection between the olfactory epithelium and glomeruli in the olfactory bulb may occur in patients with impaired olfaction due to upper respiratory tract infections since intact connectivity of olfactory sensory neurons implies enhanced nasal thallium-201 migration to the olfactory bulb in these patients. Decrease in both thallium-201 migration rate to the olfactory bulb and olfactory bulb volume are shown in patients with parosmia and normal odor recognition thresholds after upper respiratory tract infection [20]. Incomplete regeneration of olfactory sensory neurons may cause disconnection between the olfactory epithelium and glomeruli in the olfactory bulb in these patients with parosmia.

**Fig. 6:**
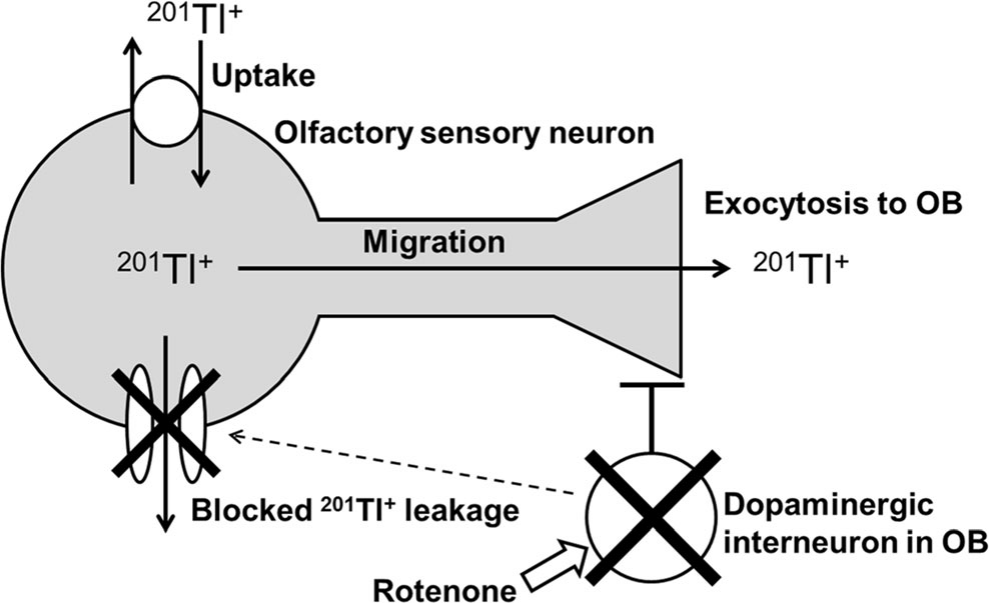
Mechanisms by which intranasal administration of rotenone induces persistent changes in intrinsic excitability of olfactory sensory neurons. In this study, because rotenone administrated to the nasal cavity was washed out in the dissection of olfactory epithelium before electrophysiological recordings, such long-lasting inhibition of ion channels by rotenone may be related with plasticity of neuronal excitability. The membrane resistance showed no degradation with the rotenone administration, indicating that the cell membrane has an insignificant damage. The intact membrane retains ^201^Tl^+^ that Na^+^/K^+^-ATPase takes into the intracellular space. Therefore, ^201^Tl^+^ spreads more toward the axon terminals of olfactory sensory neurons when administrated together with rotenone. OB, olfactory bulb

Three hours following intranasal administration of rotenone, numbers of action potentials decreased in olfactory sensory neurons only on the rotenone treated side. The reduction of firing rate may suppress leakage of intracellular ^201^Tl^+^ via voltage-gated K^+^ channels during repolarization following action potentials, because voltage-gated K^+^ channels are highly permeable to thallium [21]. In addition, membrane resistance showed no degradation with the rotenone administration, indicating that the cell membrane undergoes insignificant damage. Intact membrane retains thallium-201 that the Na^+^/K^+^-ATPase takes into intracellular space. Therefore, ^201^Tl^+^ spreads more toward the axon terminals of olfactory sensory neurons when administrated together with rotenone (Fig. 6).

Mechanisms by which intranasal administration of rotenone induced persistent changes in intrinsic excitability of olfactory sensory neurons remain unclear. One possible mechanism is the acute action of rotenone that involves modulation of ion channels through the production of reactive oxygen species [22]. However, in this study, because rotenone administrated to the nasal cavity was washed out in dissection of olfactory epithelium before electrophysiological recordings, such long-lasting inhibition of ion channels by rotenone may be related with plasticity of neuronal excitability (Fig. 6).

With the use of in vivo imaging via SPECT-CT, we demonstrated that thallium-201 migration to the axon terminals of olfactory sensory neurons was increased under reduced inhibitory input from damaged olfactory bulb interneurons, following nasal administration of rotenone in rats. Decreased thallium-201 migration rate to the olfactory bulb predicted a poorer prognosis for patients with idiopathic olfactory dysfunction [23]. Patients with idiopathic olfactory dysfunction and decreased thallium-201 migration rate to the olfactory bulb may be at high risk for pre-symptomatic idiopathic Parkinson’s disease because the overexpression of dopaminergic interneurons in the olfactory bulbs of patients diagnosed with idiopathic Parkinson’s disease has been demonstrated [24].

A limitation of this study is that we did not assess changes in thallium-201 migration rate to the olfactory bulb in mice with overexpressed olfactory bulb dopaminergic interneurons. In a current ongoing clinical trial, a thallium-201-based olfactory nerve imaging method, called olfactory scintigraphy, is under investigation with the aim of determining the thallium-201 migration rate to the olfactory bulb in idiopathic Parkinson’s disease. Chronic subcutaneous administration of rotenone induces the pathology of Parkinson’s disease in rats [25]. Therefore, farmers who experienced nasal rotenone intake during their early life may be at high risk for developing a neurological disorder similar to Parkinson’s disease. Olfactory scintigraphy that could be used to detect enhanced olfactory transport of thallium-201 could potentially allow clinicians to begin treatment sooner and prevent or lessen symptoms of a neurological disorder similar to Parkinson’s disease.

In conclusion, we demonstrated increased nasal thallium-201 migration to the olfactory bulb in mice with damage to dopaminergic interneurons in the olfactory bulb without significant damage to olfactory sensory neurons. Our findings suggested that thallium-201 migration to the axon terminals of olfactory sensory neurons was increased owing to delayed repolarization under reduced inhibitory input from damaged olfactory bulb interneurons after the nasal administration of rotenone. The thallium-201-based olfactory imaging may be useful for the diagnosis of subjects who are at risk for developing a neurological disorder similar to Parkinson’s disease. A disconnection between the olfactory epithelium and glomeruli in the olfactory bulb may occur in patients with impaired olfaction, reduced olfactory bulb volume, and nasal thallium-201 migration to the olfactory bulb, since intact connectivity of olfactory sensory neurons implies enhanced nasal thallium-201 migration to the olfactory bulb in these patients.

## Electronic supplementary material

Supplementary Figure 1 An outline of the protocol (PDF 107 kb)
