# Supplementary material for: Thallium-201 Imaging in Intact Olfactory Sensory Neurons with Reduced Pre-Synaptic Inhibition In Vivo
Source: Mol Neurobiol. 2020 Aug 20;57(12):4989–99. doi: 10.1007/s12035-020-02078-y (PMC7541386; doi:10.1007/s12035-020-02078-y)

Supplemental figure 1

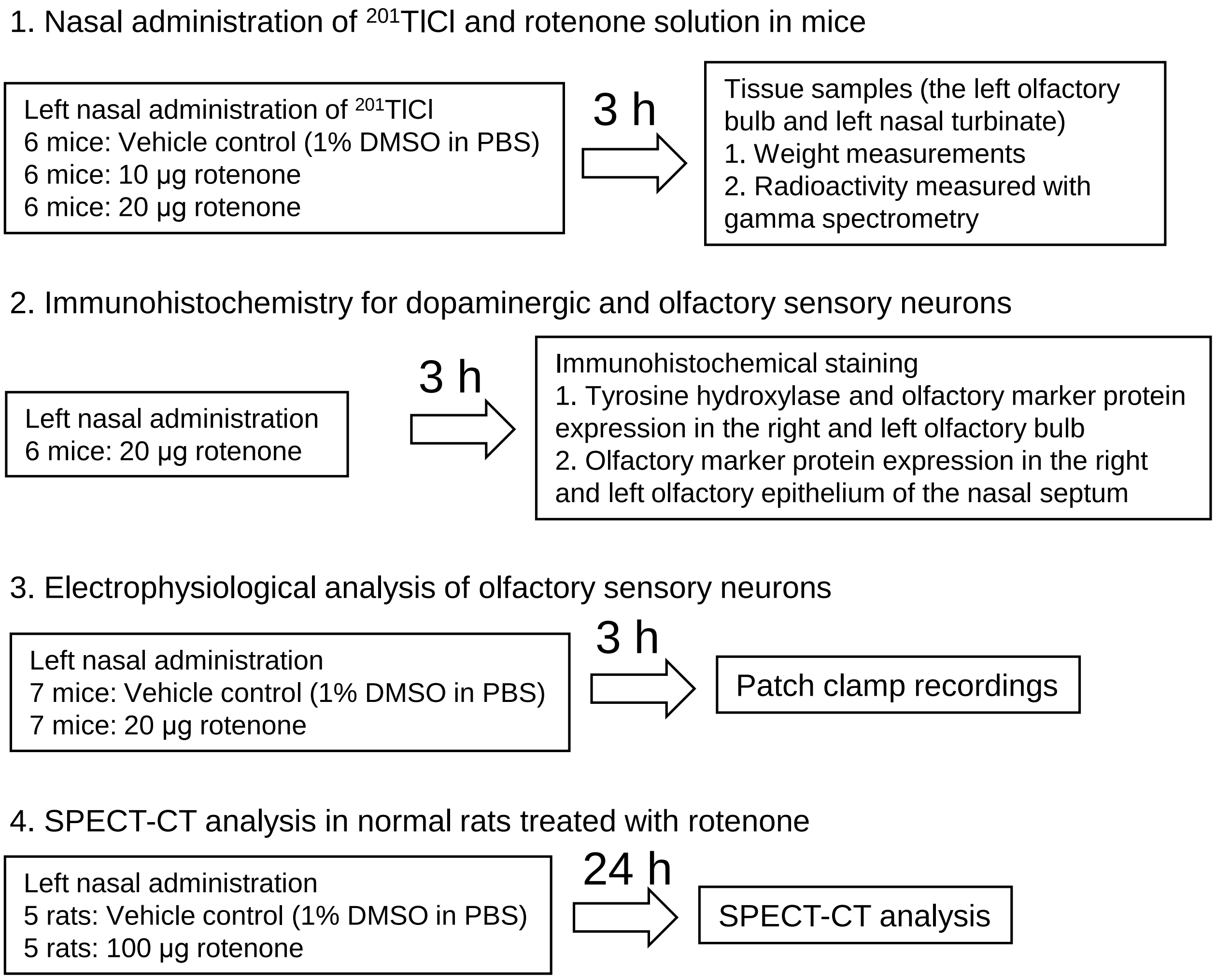

Supplement: Supplementary file 1 — An outline of the protocol (PDF 107 kb) [file 12035_2020_2078_MOESM1_ESM.pdf]
